# Hardening Effect Analysis by Modular Upper Bound and Finite Element Methods in Indentation of Aluminum, Steel, Titanium and Superalloys

**DOI:** 10.3390/ma10050556

**Published:** 2017-05-19

**Authors:** Carolina Bermudo, Lorenzo Sevilla, Francisco Martín, Francisco Javier Trujillo

**Affiliations:** Civil, Material and Manufacturing Engineering Department, ETSII-EPS, University of Malaga, Málaga 29071, Spain; lsevilla@uma.es (L.S.); fdmartin@uma.es (F.M.); trujillov@uma.es (F.J.T.)

**Keywords:** incremental forming, indentation, FEM, MUBT, plastic deformation, hardening effect

## Abstract

The application of incremental processes in the manufacturing industry is having a great development in recent years. The first stage of an Incremental Forming Process can be defined as an indentation. Because of this, the indentation process is starting to be widely studied, not only as a hardening test but also as a forming process. Thus, in this work, an analysis of the indentation process under the new Modular Upper Bound perspective has been performed. The modular implementation has several advantages, including the possibility of the introduction of different parameters to extend the study, such as the friction effect, the temperature or the hardening effect studied in this paper. The main objective of the present work is to analyze the three hardening models developed depending on the material characteristics. In order to support the validation of the hardening models, finite element analyses of diverse materials under an indentation are carried out. Results obtained from the Modular Upper Bound are in concordance with the results obtained from the numerical analyses. In addition, the numerical and analytical methods are in concordance with the results previously obtained in the experimental indentation of annealed aluminum A92030. Due to the introduction of the hardening factor, the new modular distribution is a suitable option for the analysis of indentation process.

## 1. Introduction

Indentation is generally applied in hardening tests to characterize materials [1,2]. In this study, the indentation process is studied as an incremental process like the Single Point Incremental Forming [3], the Multiple Indentation Processes [4] or the Localized-Incremental Forming Process [5], which are now being introduced in the current industry. Previous work presented the application of the new Modular consideration for the Upper Bound Theorem (MUBT) to indentation [6,7,8,9] and validated the new model with experimental tests. In this paper, the abbreviations MUBT, as opposed to Upper Bound Theorem (UBT), will be used to refer to the modular application of the method. One of the main advantages of MUBT is that the modular configuration makes possible the introduction of the parameters that are present in forming processes without overcomplicating the analysis. This fact allows an enrichment of the study, offering a closer approximation to reality. Therefore, the introduction and study of the hardening effect is considered necessary in order to get a more accurate model and approach to the current industrial processes.

Leaning on the modularity described, several Hardening Models (HMs) are established depending on the material behavior, expanding the application of MUBT. This work aims to offer a complete understanding of the HMs developed, showing their implementation in the analysis of the indentation processes for different materials. Furthermore, the approach tries to improve previous analysis, enlarging the study and adjusting the method. The modular model is presented as an analytical tool that reduces the time and cost that generally is consumed in the workpiece analysis, knowing that these workpiece analyses are usually necessary prior the final implementation of the procedures.

The UBT, under its different variants, is an analytical approach satisfactorily suitable for obtaining the necessary force to achieve plastic deformation. Some examples can be found in the studies of Moncada et al. [10], where a special case of ring compression test with non-symmetrical neutral plane of material flow is analyzed under the UBT perspective. In addition, Yunjian et al. [11,12] present an upper bound solution of axial metal flow for rods and later, an upper bound model for strain inhomogeneity analysis in radial forging processes. Alforzan and Gunasekera [13] use the UBT as an elemental technique to design axisymmetric forging by forward and backward simulation. On the study of the indentation process, the Triangular Rigid Zone (TRZ) alternative is the kinematic-geometrical option that allows a more accurate solution, with a greater capacity of analysis, as shown in the work of Kudo [14] and, recently, proved again by Topcu [15].

**Figure 1:**
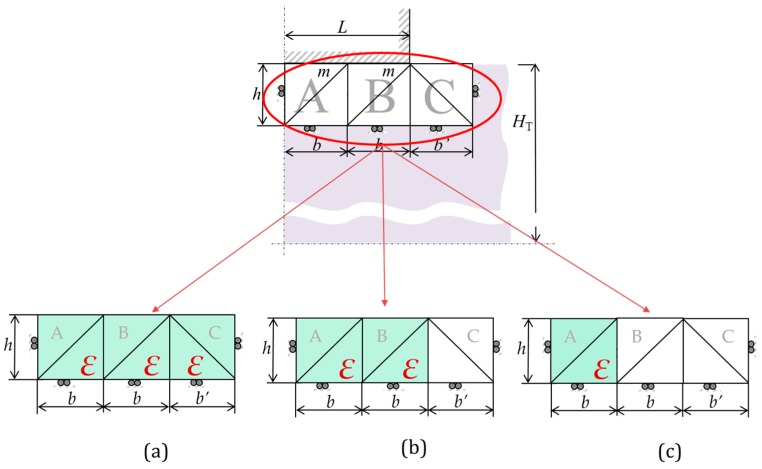
Optimal MUBT model (three modules with two triangular rigid zones each) and hardening effect (ε) distribution for each Hardening Model (HM): (**a**) HM1; (**b**) HM2; and (**c**) HM3.

Focusing on its modular application, the optimal modular model contemplates the material flow that exists under the punch and near it (Figure 1). This area of the material suffers more from the stresses and strains that occur during the forming process. The model implemented consists of 3 modules with 2 Triangular Rigid Zone (TRZ) each. The modular concept gives a better approximation to the process, allowing the inclusion of more modules if necessary. In this case, after the study of the optimal number of modules, it was demonstrated that a 3-module model offers lower forces. The study was performed taking into account different forming parameters as friction or hardening [16]. Figure 1 shows the module distribution, where *L* is half the width of the punch, *m* is the friction coefficient, *b* is the base of module A and B, *b’* is the base of module C, *H_T_* is the height of the quarter of the workpiece analyzed, *h* is the height of the modules and *V* is the punch speed. A double symmetry is imposed to simplify the analysis. The double symmetry makes possible to focus only on a quarter of the workpiece. In addition, the proportion established between the punch and the workpiece is considered as an infinite analysis. Thus, the symmetrical punch under the workpiece can be ignored. The forces obtained for the punch above the workpiece are the same as the forces for the symmetrical punch. The analysis becomes equivalent to forming process with only one indenter like the Single Point Incremental Process or the Multiple Indentation Forming Process. 

**Figure 2:**
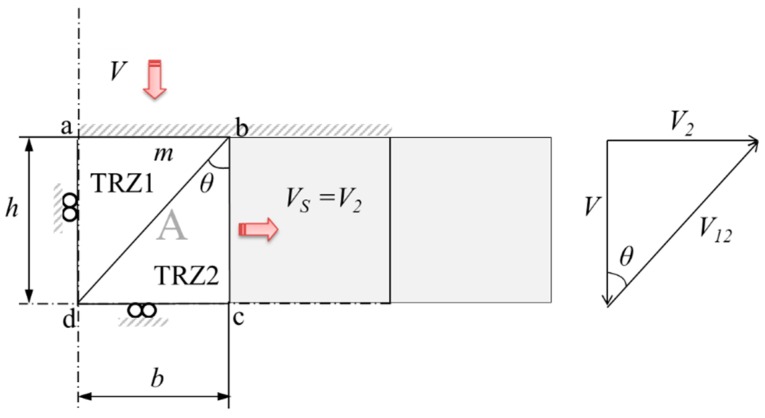
Module A analysis and velocity hodograph.

Thanks to the modular configuration, each module can be individually analyzed, obtaining an easier *p*/2*k* relations [17], being *p* the effort required to deform the work-piece and *k* the shear yield stress. After the hodograph for module A is completed (Figure 2), the (*p*/2*k*)_A_ relation is shown in Equation (1), obtained from the UBT general expression [10].

(1) $$p\times b\times V\times w=k\times w\times \left[V_{12}\times bd+V_{2}\times dc\right],$$

Following the diagram in Figure 2, Equation (2) is obtained:

(2) $${\left(\frac{p}{2k}\right)}_{A}=\frac{1}{2\times b}\times \left[\frac{h^{2}+2b^{2}}{h}\right],$$

Accordingly, the same analysis is made for the other two modules. Finally, a weighted average is applied to obtain the final *p/2k* values:

(3) $${\left(\frac{p}{2k}\right)}_{T}=\frac{{\left(\frac{p}{2k}\right)}_{A}\times b+{\left(\frac{p}{2k}\right)}_{B}\times b+{\left(\frac{p}{2k}\right)}_{C}\times b^{\prime }}{2\times b+b^{\prime }},$$

The simplified Ludwik equation (Equation (4)) is used for the introduction of the hardening effect in the specific indentation case study, considering always plane strain conditions and cold-worked metals.

(4) $$\sigma =Y+K\times \epsilon^{n},$$

where *Y* is the yield strength, *σ* is the stress analyzed, *K* is the strength coefficient that depends on the material structure, and *n* is the strain hardening exponent, also specific for each material. 

However, Equation (1) is expressed in terms of the yield stress (*Y*) and the true strain (*є*). Working with the modular model, a transformation must be performed in order to adapt its application. MUBT studies deformation instants. Therefore, an approximation to the engineering strain (*e*) is necessary to be able to work with the original length and area. True and engineering strains are calculated as follows:

(5) ϵ=ln(1+e),

where, assuming the Von Mises yield criteria:

(6) 2k=1.155Y,

(7) k=0.577Y,

Thus, the equation applied in the study of the hardening effect implementation in MUBT, regarding an indentation process, is:

(8) $$k=k_{0}+0.577\times K\times \epsilon^{n},$$

with *k* being the yield stress in shear at that analyzed instant and k_0_ the previous yield stress in shear. To validate the MUBT application considering different materials, a numerical study is performed using the Finite Element Method (FEM). This method allows the introduction of a considerable number of parameters for the proper study of plastic deformation, being the hardening effect among them. Thus, an indentation model is implemented to simulate the process over different materials and alloys. Previous analysis with aluminum A92030 [8] showed that MUBT was able to offer results close to reality. Present work implements a numerical simulation to consider a more varied range of materials and validate the HMs developed. 

Thus, this study focus on the analysis and application of the HMs developed for indentation, verifying its application through a numerical analysis. With the numerical analysis, a wide range of materials is analyzed, supporting the validation of the model developed and expanding its application. 

## 2. Materials and Methods 

### 2.1. Finite Element Analysis 

To validate the MUBT application for an extensive number of materials, a series of simulations is performed with DEFORM 2D (version 8.1, Scientific Forming Technologies Corporation, Columbus, OH, USA) [18]. This FEM software allows the implementation of different materials from its own material database. In this case study, the materials and alloys considered are aluminum, steel, titanium, and superalloys.

Steel, aluminum and their alloys have been widely introduced in the industry due to their applications. Within the aluminum and steel group, the materials chosen from the database for the FEM study are presented in Table 1. Materials are named under the Unified Numbering System (UNS) codification.

Table 1 only shows the materials selection obtained directly from the DEFORM 2D data base. Materials that are not offered within this data base can be incorporated with the manual introduction of their properties values, like the simulation carried out with the annealed aluminum A92030 used in the experimental tests.

Likewise, working with titanium and superalloys is interesting. These materials usually have excellent mechanical strength and good resistance to creep at high temperatures. They also exhibit good resistance to corrosion and oxidation [19,20]. Within the titanium and superalloy group, the materials chosen are presented in Table 2. Even though the indentation processing of superalloys is unusual, the analysis is conducted as an extension of the study, showing the versatility of the developed model and opening the field to their consideration in further incremental processes.

Due to its importance and presence in the industry, the study of the indentation process for the materials in these four groups was established. All the materials were simulated with FEM and the results obtained were compared with the modular model developed. The correlation of both methods is shown in the next section. 

**Figure 3:**
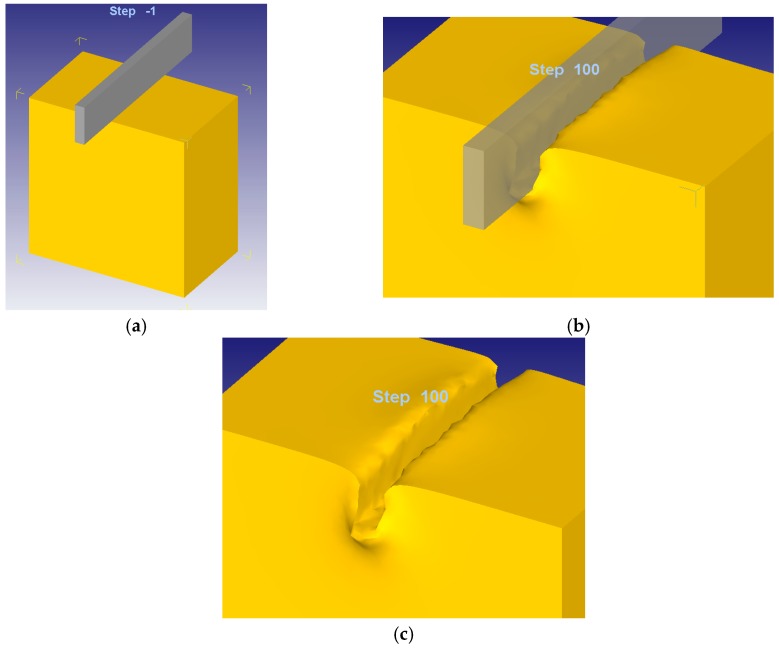
A 3D FEM model of the 50 × 50 × 30 mm sample and indenter: before indenting (**a**); during indentation process (**b**); and workpiece after indentation (**c**).

For the boundary conditions set in the FEM simulation, an infinite case study has been considered. Although the finite and infinite cases have been covered in previous studies [21], the finite consideration is far from the indentation analyzed in this work, resembling processes such as stamping or shearing. Therefore, in this paper, only the infinite consideration will be taken into account. Figure 3 shows the 3D model implemented with FEM.

**Figure 4:**
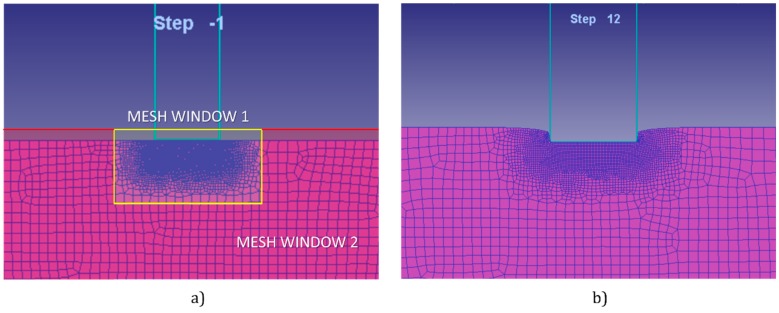
Mesh windows distribution: before indentation (**a**); and during indentation (**b**).

In previous studies [6], the geometry of the punch was studied under the same FEM software (version 8.1, Scientific Forming Technologies Corporation, Columbus, OH, USA). A mesh analysis was carried out in order to found the optimal mesh that will offer accurate solutions. Working with numerical methods, it is important to implement a mesh that gives solutions close to reality without increasing too much the resolution time. Accurate solutions in reasonable time can be achieved implementing mesh windows. The mesh widows allow an analysis with a high density mesh in the zone near the punch, were the deformation is taking place, leaving the rest of the workpiece with a coarse mesh. The mesh window (6 × 4 mm approximately) is located in the area where the punch performs and establishing and adequate relative element size of 1/20. That is to say that the elements placed inside the coarse mesh are 20 times larger (Figure 4). In addition, to avoid the distortions of big elements, a remesh has been established every three steps. 

The elements used for the FEM analysis are two-dimensional plain strain elements of four nodes. Vertical displacements are fixed at the bottom of the sample as boundary conditions, without considering friction for the restriction. An elasto-plastic isotropic hardening model has been used as constitutive material model.

For the contact between the workpiece and the punch surface a shear type friction for could forming has been defined with a value of 0.12.

For each material simulated, the flow stress law attends as the form $\bar{\sigma }=\bar{\sigma }\times \left(\bar{\epsilon },\dot{\bar{\epsilon }},T\right)$, where $\bar{\sigma }$ is the flow stress, $\bar{\epsilon }$ is the effective plastic strain, $\dot{\bar{\epsilon }}$ is the effective strain rate and *T* is the temperature.

Finally, the simulations are set for 100 simulations steps, saving every five steps, with a maximum remesh increment of 3 steps and with an equal punch displacement of 0.06 mm. 

Knowing that the present work is intended as a continuation of the implementation and validation of the hardening models developed for MUBT, the experimental indentation carried out with annealed aluminum A9230 is additionally introduced.

### 2.2. Experimental Test 

Test are carried out with a universal tension-compression machine (Servosis, Madrid, Spain), Servosis ME 405 [22], which applies continuous compression force with a maximum load capacity of 100 kN. Working with materials that deform within that deformation range is essential. The force range of the tension-compression machine is limited and deep indentations were not possible. With the annealing process on the aluminum A92030, the material allows deformations with lower forces, permitting deeper penetrations. Hence, working with the materials shown in Table 1 is not possible due to the limitations present in the equipment available. Notwithstanding, the material tested, aluminum A92030 after the annealing process, was manually introduced in the software database in order to be able to work with it during FEM simulations. 

For the indentation, a 3 mm width punch made of steel AISI 304 is used. The aluminum A92030 workpiece is obtained from a 50 × 50 × 2000 mm square section bar (Table 3). 

To achieve plane strain conditions, the workpiece depth is 10 times the wide of the punch [23]. Thus, the workpieces final size is 50 × 50 × 30 mm.

**Figure 5:**
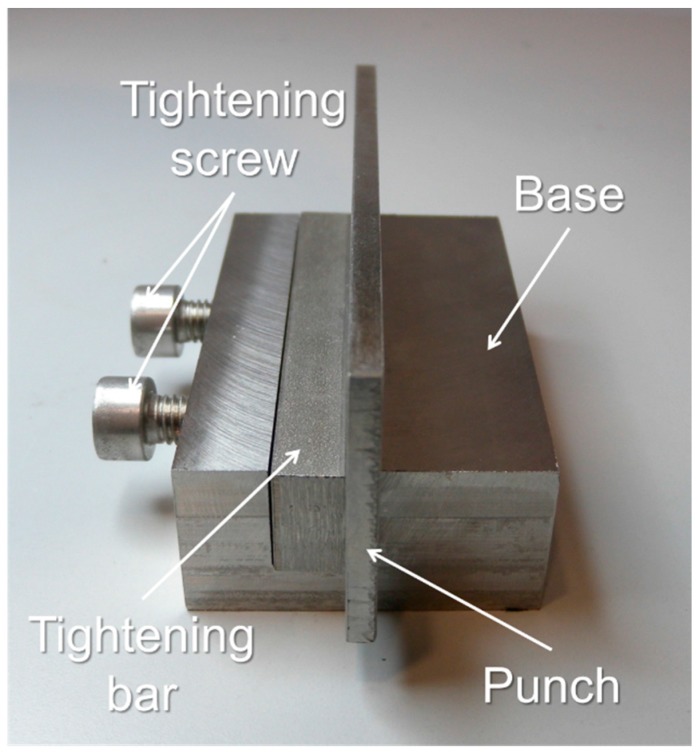
Assembled punch fastening tool.

A tool is designed to avoid the inclination of the punch (Figure 5). The system provides a wider base for the indenter.

**Figure 6:**
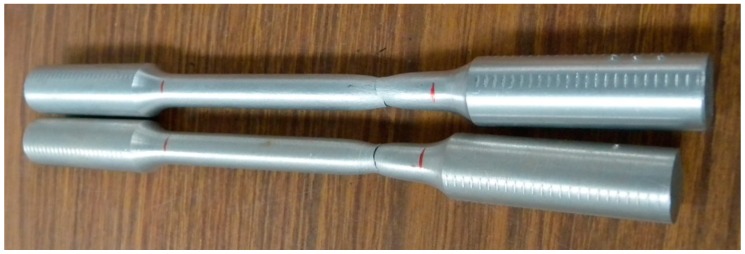
Tested A92030 tensile samples.

The aluminum A92030 is subjected to a controlled annealing treatment, to attain an adequate depth during the indentation tests. After the annealing process, tensile test (UNE-EN ISO 6892-1 [24]) (Figure 6) were conducted to obtain the strength coefficient *K* and the strain-hardening exponent *n* of the annealed aluminum. The values obtained were *n* = 0.26 and *K* = 404.66 MPa.

An indentation of 6 mm was conducted. The overcoming of the tensile strength limit is not intended, so tests were stopped when cracks start to appear in the workpiece. 

Twelve tests were performed, three for every speed range (0.6 mm/min, 4 mm/min, 60 mm/min and 400 mm/min), to analyze the influence of the speed in the hardening effect. Due to the correlation between the tests performed in the same range of speed, there was no need to increment the number of experiments. The DOE tool for experimental validation was also considered but, being non-complex tests and not having a large number of input or output variables, its application was not necessary. Each sample was given a code for identification, as follows:

E*X*_1_-*X*_2_-*X*_3_-*X*_4_-*X*_5_

where

*X*_1_: Specimen number;
*X*_2_: Speed (mm/min);
*X*_3_: Indentation depth (mm);
*X*_4_: Specimen material (A: Annealed); and
*X*_5_: Specimen dimension (mm).

## 3. Results and Discussion

Three hardening models were developed. Due to the experimental tests performed with annealed aluminum A92030, the FEM model was validated, allowing the simulation of a wider range of materials. 

Finding a behavior pattern within each metal group was essential. This was possible due to the comparisons between FEM and MUBT. Being the strain hardening exponent (*n*) a measure of the material hardening during the forming process, it is established as a categorization parameter. The hardening models are classified according to *n.* For the different materials analyzed with FEM, the ASTM 646 standard is applied for the *n* calculation, knowing that for ductile materials at room temperature, typical values are between 0.02 and 0.5 [25,26].

The numerical application used provides only the flow stress data, i.e., the data for a material in the plastic region. Furthermore, these data represent the true stress-strain curve, making it possible to obtain the necessary information to deduce *n* and *K* for each simulated material. These results are also shown in Table 1 and Table 2. Thus, a classification was established according to *n* (Table 4). Superalloys, due to their special characteristics, needed to be grouped separately.

The modular model is compound of three different modules. Therefore, according to Table 2, three HM can be considered. The HM1 (Figure 1a) contemplates that all the material under the punch suffers the hardening effect. Therefore, the hardening equation (Equation (8)) is applied to the three modules, two of which are located below the punch (A and B) and the third outside the punch area (C). In this case, the third module that is not under the punch, experience deformation due to the push of the material under the punch and, therefore, will also experience strain hardening.

The HM2 (Figure 1b) only applies the hardening effect to the modules located under the punch. In this case, the assumption that module C does not suffer the same deformation as module A and B was made. Thus, hardening for module C may be negligible. The material considered in the modules directly under the punch receives all the compressive force. Module C is proposed with the ability to absorb this material displacement. That is to say, the hardening effect of module C could be neglected in relation to the hardening behavior that the preceding modules suffer.

**Figure 7:**
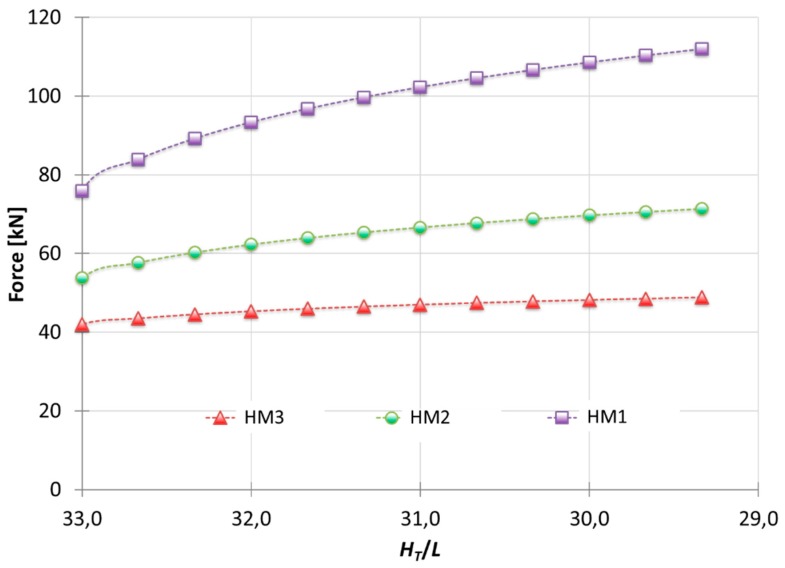
Results for Aluminum A92030 when the different Hardening Models apply.

For the HM3 (Figure 1c), only module A suffers from hardening. This model is suitable for materials which briefly harden due to deformation, that is, materials with small *n* (between 0.05 and 0.10). Therefore, the hardening effect will be concentrated only in module A, leaving the remaining modules without it. Figure 7 illustrates the difference between the three hardening models applied on the analysis of A92030, for different shape factors (*H_T_*/*L*, being *L* constant along the graphic evolution). It can be seen that when the HM1 is implemented, the forces obtained are much higher than in the case study with the HM3. This is due to the considerations of the hardening effect module by module. The HM1 considers *ε* in all of the modules versus the HM3 that only implements *ε* in the first module, with the intention of simulate the materials with less hardening under a plastic deformation. 

As can be seen in Figure 1, the shape factor is the quotient between the total height of the quarter of the workpiece studied and half of the indenter length.

Materials presented in Table 1 and Table 2 were simulated with FEM and studied with MUBT, solving each material with the HM1, HM2 and HM3. The classification presented in Table 4 was established through the comparison between MUBT and FEM. The HM3 offered accurate results for materials with a hardening exponent under 0.1. With the HM1 and HM2, the effort obtained were over the FEM range because more hardening effect was being considered. In addition, for materials with *n* over 0.1, the results obtained from the HM2 were of the same range as the results from the FEM, while the results from the HM3 were lower, having a higher error percentage.

Next, some examples are illustrated to visualize the proper fitting between the numerical analysis and analytical solution with the hardening effect implemented. The hardening models follow the same evolution within their corresponding group (HM1, HM2 or HM3). All the materials presented in Table 1, Table 2 and Table 4 were simulated but due to the similar progress of the results provided graphically for each group, only part of them are presented in this paper in order to avoid similar images. Preceding studies [9,17] present a previous analysis of the case study. After an improvement of MUBT and the FEM model, results show more accurate solutions, reducing the difference between the effort values and a better concordance with each other. In addition, the results are plot only focusing on the infinite case study, avoiding higher *H_T_*/*L* values. 

### 3.1. Results for HM3

**Figure 8:**
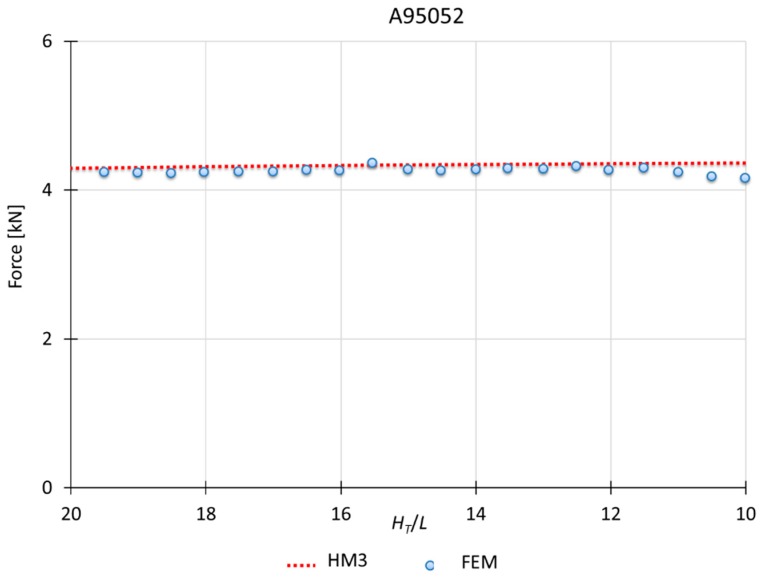
MUBT-FEM comparison for A95052, *n* = 0.09.

**Figure 9:**
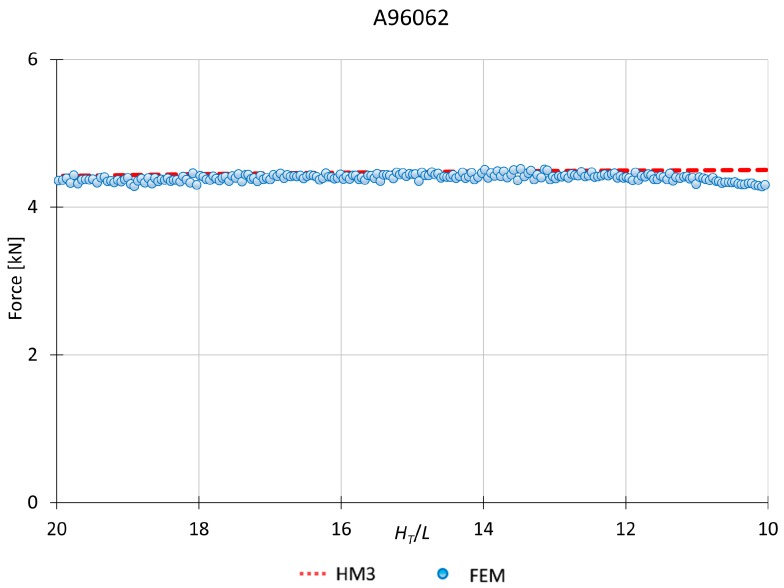
MUBT-FEM comparison for A96062, *n* = 0.10.

This model applies to materials which *n* is between 0 and 0.10. Therefore, the hardening effect is only considered in the first module (module A). Figure 8 and Figure 9 show a MUBT-FEM comparison for two different materials, aluminum A95052 and steel G10450, being *H_T_*/*L* the shape factor. In addition, the workpiece simulated is higher than the one used for the experimental tests due to the possibility to analyze an infinite case with a bigger sample in FEM. 

It can be seen how MUBT results present a close approximation to those given by FEM, establishing that HM3 is suitable for materials with hardening exponent between 0.05 and 0.10. With the implementation of the mesh windows and increasing remesh parameters, FEM results offer values with less alterations, like the pick that usually can be obtained due to a thick mesh or lower remesh criteria. The FEM study is carried out for plane strain conditions. The software used considers a workpiece depth of 1 mm. That will explain the low force values obtained for the different materials simulated.

Figure 9 shows the approximation between MUBT and FEM for Aluminum A96062. In this case, FEM results have not been depurated in order to present all the values that can be obtained due to the increase in the remesh criteria. After depuration, the results are offered as can be seen in the rest of the plots represented. This depuration is necessary to present clear results and be able to make an accurate comparison between methods.

### 3.2. Results for HM2

**Figure 10:**
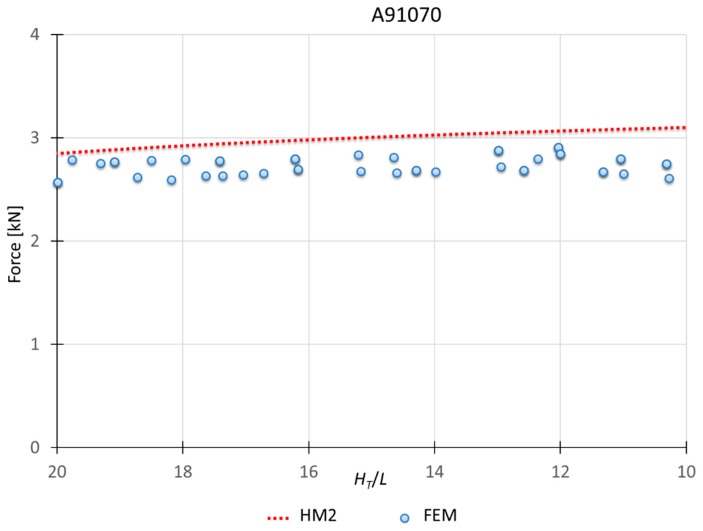
MUBT-FEM comparison for A91070, *n* = 0.21.

**Figure 11:**
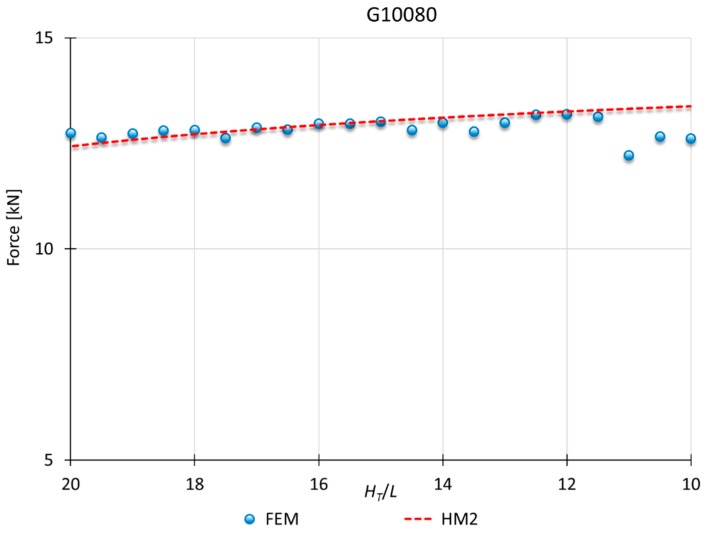
MUBT-FEM comparison for G10080, *n* = 0.17.

**Figure 12:**
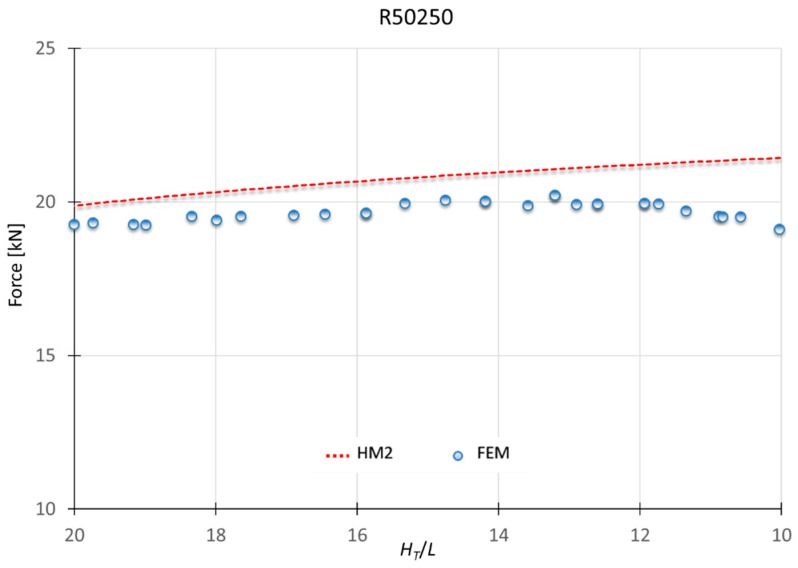
MUBT-FEM comparison for R50250, *n* = 0.23.

This model applies to materials which *n* is greater than 0.10. Therefore, the hardening effect is considered in the modules under the punch (module A and B). As seen in Figure 7, to consider the hardening effect for all the modules (HM1) show results excessively far from the results obtained for this type of materials. Figure 10, Figure 11 and Figure 12 show a MUBT-FEM comparison for aluminum A91070, steel G10080 and Titanium R50250 with *n* 0.21, 0.17 and 0.23 respectively.

Again, a close approximation is obtained. Although a small difference is visible, this difference is not higher than 15%, which is consistent with other UBT studies [27,28].

### 3.3. Results for HM1

**Figure 13:**
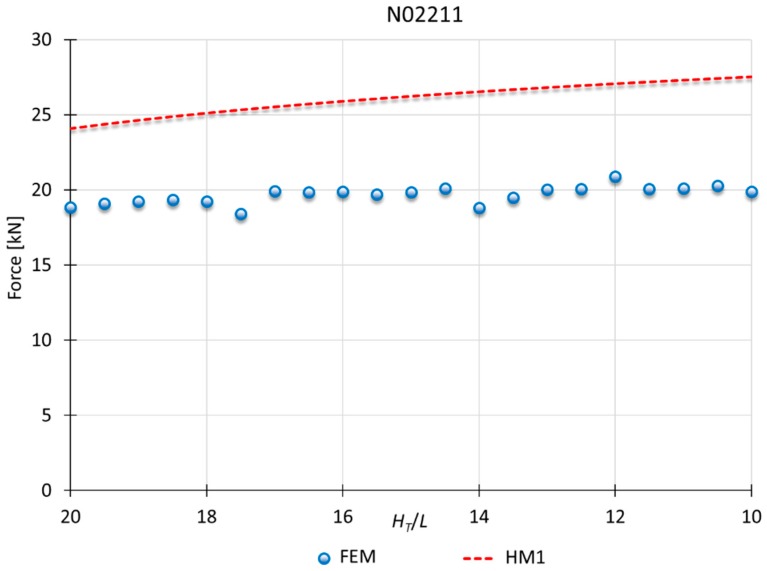
MUBT-FEM comparison for N02211, *n* = 0.21.

**Figure 14:**
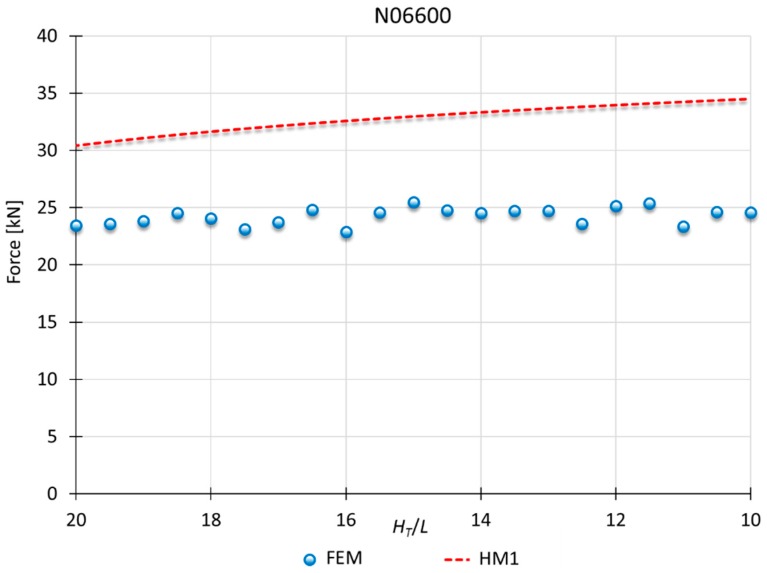
MUBT-FEM comparison for N06600, *n* = 0.20.

Finally, the comparison between MUBT and FEM for superalloys is shown in Figure 13 and Figure 14 for nickel N02211 and Inconel N06600, respectively. For these materials, HM1 presents a better fit.

In this case, HM1 presents results more detached from those obtained with FEM. Notwithstanding, the results are consistent with the error percentage shown in other studies that UBT [29,30,31,32].

In addition, superalloys can be considered extreme cases, being out of the range of the present analysis. These types of materials are not usually used in cold indentation processes, being processed by vacuum induction melting, investment casting, powder metallurgy or spray forming/casting [33,34,35]. Superalloys need special treatment asides from other metals, being necessary a sub-classification and new adjustment of the model due to their behavior under great deformation. The study opens the field to the consideration of processing superalloys with this kind of process and its analysis with MUBT.

### 3.4. Experimental Results

**Figure 15:**
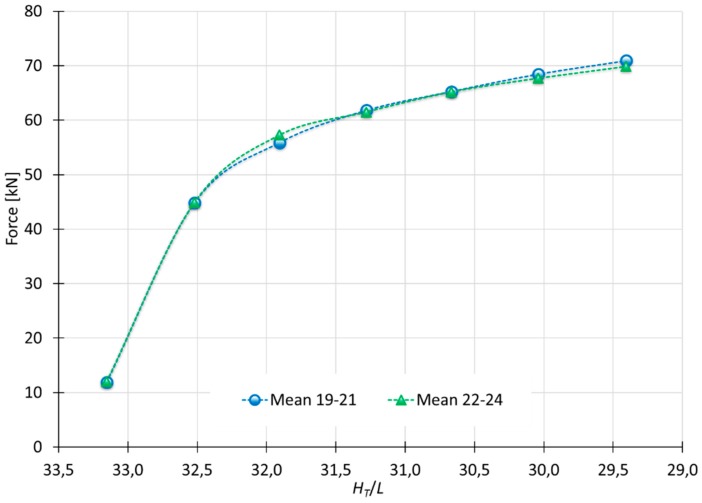
Indentation forces obtained from test with different speeds.

Figure 15 shows the mean values of the tests E19/E20/E21-4-1.5-6-Al92030A-50 × 50 × 30 and E22/E23/E24-60-1.5-6-Al92030A-50 × 50 × 30. According to these results, speed does not produce significant changes in the final loads obtained [8]. 

**Figure 16:**
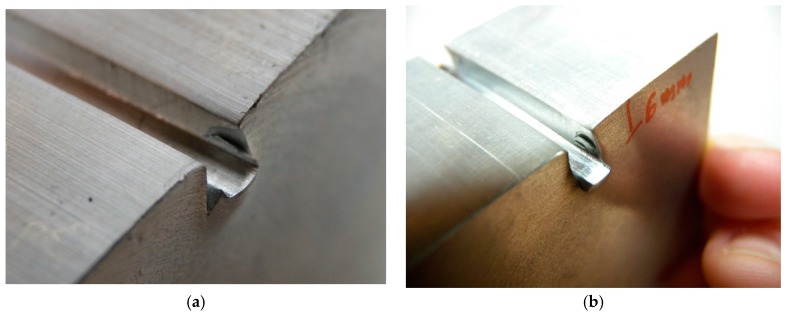
Cracks near the indenter boundary on specimen: E17 (**a**); and E23 (**b**) after indentation.

Figure 16 shows the indentation achieved for samples E17 and E23. Certain fragility in the material is appreciated near the margins of the punch. Cracks appear in the surface of the work-piece. This phenomenon justifies that deep indentations are undesirable due to possible distortions on final results.

**Figure 17:**
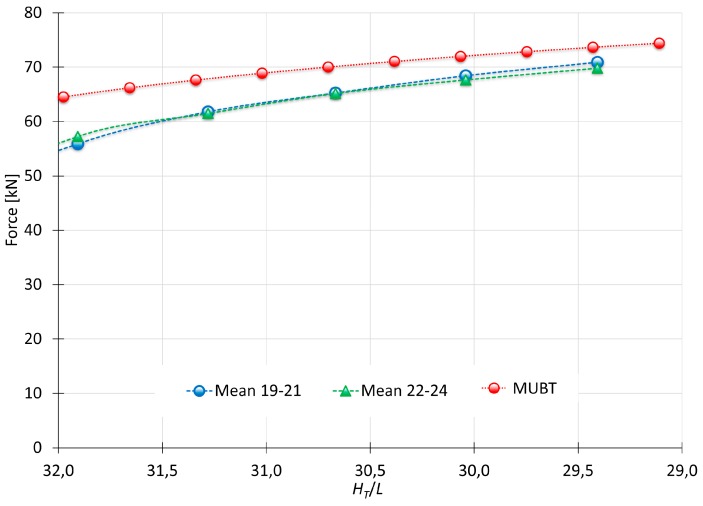
Comparison between MUBT and Experimental tests.

The comparative between the performed tests and MUBT (Figure 17) shows that the model follows the same evolution as the results obtained from the indentation tests carried out. 

As the punch penetrates, differences between MUBT and the experimental tests decreases. Considering this study focuses on new incremental processes (Single Point Incremental Process, Localized-Incremental Forming Process or the Multiple Indentation Forming Process mentioned previously) that are being developed nowadays, shallow indentations (corresponding to the first values of the graph) can be disregarded. Hence, with a 2 mm indentation, the difference between MUBT and the test at 4 mm/min and 60 mm/min are 11.4% and 8.8%, respectively. In addition, at a 6 mm indentation, the differences are 0.6% and 2.1%, respectively, very close values that show an optimal adaptation of MUBT. 

## 4. Conclusions

Present work shows the development of the hardening models implemented in MUBT. Three HMs were analyzed in order to achieve reasonable fitting considering different materials. The model classification is established through the hardening exponent (*n*). Different simulations applying numerical methods were carried out and compared with the results obtained with MUBT.

For the metals taken into account in the case studies, superalloys needed special treatment. This type of metals presents a special behavior since they do not follow the common tendencies of other regular metals. Consequently, the adjustment of the *n* categorization for superalloys is considered normal. 

The results obtained from the hardening models developed are consistent with other analysis performed, like FEM or experimental validation with aluminum A92030. The loads obtained with the numerical method and tests are compared with those procured by MUBT, displaying a set of results in the same range.

For the *n* values from 0 to 0.10, where HM3 applies, MUBT results evolve similarly to the simulation results. The results only show a difference between the 3% and the 5%. For the *n* values above 0.10, where HM2 applies, the modular model, the numerical analysis and the experimental validation (the aluminum tested has a *n* = 0.26) also display loads with a good correlation, with differences between the 5% and the 10%, depending on the shape factor (*H_T_*/*L*). In general, the model tends to stabilize, showing a progressively approach to the real load values.

Only the superalloys, for which HM1 applies, present a bigger disparity in the results. However, the superalloys case is presented as an extension of the analysis. This extension shows the capacity and versatility of MUBT. It exposes the possibility of a new subcategorization for the superalloys group to better adapt the model to their evolution. Notwithstanding, the results obtained are within the acceptable range according to other studies in the application of the Upper Bound Element Method referenced in this paper work. In addition, these types of materials are not usually used in cold indentation processes, being processed by vacuum induction melting, investment casting, powder metallurgy or spray forming/casting. Thus, the difference between the results obtained is not relevant in this case study, but opens the field to the consideration of processing superalloys with this kind of process.

Therefore, this paper is presented as a further step in the validation of the MUBT application to an indentation process considering the hardening effect, showing once more the suitability of the model and the accuracy of the results that can be obtained working with conventional materials. The investigation offers three hardening models that permit the study of a wide range of materials (aluminum, steel, titanium, etc.). The delimitation of the three hardening models allows obtaining results in concordance with preceding studies and close to reality foe each material.

## Figures and Tables

**Table 1:** Aluminum and steel alloys characteristics. Unified Numbering System codification (UNS).

Aluminum				Steel			
Code	*Y* (MPa)	*K* (N/mm^2^)	*n*	Code	*Y* (MPa)	*K* (N/mm^2^)	*n*
A92024	270	366.44	0.2	G10450	640	881.83	0.10
A95052	140	192.48	0.09	G10080	280	577.67	0.17
A96062	138	198.54	0.10	S30400	510	1073.84	0.19
A93003	120	199.54	0.12	S30200	250	1055.74	0.42
A96082	200	355.29	0.11	S32100	501,80	1084.64	0.28
A91070	68	130.45	0.21	-	-	-	-

**Table 2:** Titanium and superalloys characteristics. UNS codification.

Superalloys				Titanium			
Material	*Y* (MPa)	*K* (N/mm^2^)	*n*	Material	*Y* (MPa)	*K* (N/mm^2^)	*n*
N06600	434.37	1101.03	0.20	R58010	1050	1758.12	0.17
G52986	762.62	1124.39	0.12	R50250	510	951.46	0.23
N02211	337.84	896.05	0.21	R50400	850	1398.28	0.20
G33106	660	888.91	0.008	R53400	1192,79	1315.54	0.02
-	-	-	-	R50250	510	951.46	0.23

**Table 3:** A92030 (UNS classification) composition.

Al (%)	Cu (%)	Pb (%)	Mg (%)	Mn (%)	Others
90.5	3.9	1.2	0.9	0.8	Rest

**Table 4:** Classification according to *n* values.

Material	*n*	HM
Aluminum, steel, Titanium and its alloys	0 ≤ *n* ≤ 0.10	HM3
	*n* > 0.10	HM2
Superalloys	0 ≤ *n* ≤ 0.12	HM3
	*n* > 0.12	HM1
